# Initiation and progression of α-synuclein pathology in Parkinson’s disease

**DOI:** 10.1007/s00018-022-04240-2

**Published:** 2022-03-26

**Authors:** George K. Tofaris

**Affiliations:** 1 Nuffield Department of Clinical Neurosciences, University of Oxford, Oxford, UK; 2 Kavli Institute for Nanoscience Discovery, University of Oxford, Oxford, UK; 3 Department of Neurology, John Radcliffe Hospital, Oxford, UK

**Keywords:** Neurodegeneration, Fibril, Oligomers, Strains, Lewy body, Propagation

## Abstract

α-Synuclein aggregation is a critical molecular process that underpins the pathogenesis of Parkinson’s disease. Aggregates may originate at synaptic terminals as a consequence of aberrant interactions between α-synuclein and lipids or evasion of proteostatic defences. The nature of these interactions is likely to influence the emergence of conformers or strains that in turn could explain the clinical heterogeneity of Parkinson’s disease and related α-synucleinopathies. For neurodegeneration to occur, α-synuclein assemblies need to exhibit seeding competency, i.e. ability to template further aggregation, and toxicity which is at least partly mediated by interference with synaptic vesicle or organelle homeostasis. Given the dynamic and reversible conformational plasticity of α-synuclein, it is possible that seeding competency and cellular toxicity are mediated by assemblies of different structure or size along this continuum. It is currently unknown which α-synuclein assemblies are the most relevant to the human condition but recent advances in the cryo-electron microscopic characterisation of brain-derived fibrils and their assessment in stem cell derived and animal models are likely to facilitate the development of precision therapies or biomarkers. This review summarises the main principles of α-synuclein aggregate initiation and propagation in model systems, and their relevance to clinical translation.

## Introduction

Parkinson’s disease (PD) is the second most common neurodegenerative disease. It is characterised by the motor symptoms of tremor, rigidity, bradykinesia, and postural instability as well as non-motor symptoms such as constipation, postural hypotension, REM sleep behavioural disorder, apathy, and dementia. PD manifests differently in each patient but three broad sub-types are well recognised: those with tremor-dominant, unilateral, and slowly progressive disease; those with symmetrical motor disease, poor cognition, REM sleep behavioural disorder, postural hypotension and relatively fast progression and those with intermediate symptoms [1, 2]. PD has a long prodromal phase that is often associated with non-motor symptoms such as REM sleep behavioural disorder. Despite the diversity of the symptoms and the heterogeneity of the clinical presentation, aggregation of α-synuclein along relevant neuronal networks in the autonomic nervous system, brainstem, and cortex has provided a unifying molecular mechanism for therapeutic intervention in PD [3]. Aggregation of α-synuclein in oligodendrocytes is the neuropathological feature of a related but more aggressive parkinsonian condition termed Multiple System Atrophy, MSA [4] and diffuse deposition of α-synuclein inclusions in the cortex and brainstem is the neuropathological hallmark of Lewy body dementia [5]. Neuronal α-synuclein aggregates are also detected in cases of Alzheimer’s disease and rarer neurogenetic conditions [6–8].

The causative link between α-synuclein and disease is supported by (a) the identification of mutations or multiplications in the α-synuclein gene (*SNCA*) in familial PD and polymorphisms in the *SNCA* locus as the commonest risk in sporadic PD [9]; (b) the propensity of monomeric α-synuclein to self-assemble into filaments, that resemble by electron microscopy those extracted from PD brain [4, 5] and (c) the evidence that increased expression or aggregation of α-synuclein in animals causes neuronal dysfunction or degeneration [10]. Neurodegeneration results from the formation of α-synuclein assemblies that are seeding-competent, i.e. able to template further aggregation, and toxic at least partly by interfering with synaptic vesicle recycling and organelle homeostasis.

Although intraneuronal α-synuclein aggregates termed Lewy bodies or Lewy neurites constitute the defining neuropathological feature of PD at post-mortem, these inclusions per se are only the visible tip of the “pathological iceberg”. Indeed, Lewy bodies at post-mortem do not always correlate with the disease severity in patients [11, 12]. It is important to note that beyond the Lewy pathology that is detected by classic immunohistochemical staining, α-synuclein aggregates are widespread and especially abundant in neuronal terminals when visualised with more sensitive techniques [13]. Therefore, the early stages of α-synuclein aggregation are likely to be the most damaging whereas inclusions may represent the end-stage process of disposing larger assemblies of misfolded proteins. Understanding the molecular mechanisms of these neuropathological changes and reproducing them in the laboratory has opened the way for rational targeted therapies and biomarkers. A number of comprehensive reviews are available on the structural plasticity and pathogenicity of α-synuclein [14, 15]. Here, I discuss past and recent studies that elucidated the principles of aggregate initiation and propagation in model systems, their key molecular underpinnings and their relevance to clinical translation.

## Initiation of α-synuclein aggregation entails aberrant membrane interactions

α-Synuclein is one of the most abundant neuronal proteins accounting for 0.5–1% of total brain protein and it is enriched in presynaptic terminals, giving the typical punctate neuropil pattern in the normal brain [16, 17]. In presynaptic terminals its function is not essential at least in animals when genetically knocked out, causing only subtle deficits such as an activity-dependent negative regulation of neurotransmission [18] or impaired dilatation of the exocytic fusion pore [19]. Instead, the pathogenicity of α-synuclein is due to a toxic gain-of-function when mutated or overexpressed. Even modest overexpression of α-synuclein in animals, in the range predicted for *SNCA* gene multiplication in patients, reduced synaptic vesicle density at the active zone and impaired the reclustering of synaptic vesicles after endocytosis without detectable neuropathology [20]. Given its physiological localisation and abundance, it is reasonable to consider the synaptic terminals as the sites of initiation of α-synuclein pathology in PD. This was first supported by animal models where transgenic expression of the aggregation-prone C-terminally truncated α-synuclein or viral expression of the full-length protein led to aggregate formation and impaired dopamine storage and release without overt neuronal death [21, 22], at least partly by redistribution of the synaptic SNARE proteins [23]. Soluble oligomers of α-synuclein block SNARE-dependent vesicle lipid mixing in vitro*,* suggesting a mechanism by which initial aggregation events may impair synaptic vesicle fusion with the plasma membrane [24, 25]. These early synaptic changes in response to α-synuclein aggregation may occur at a potentially reversible phase of the disease as shown in animals where suppression of α-synuclein expression led to partial clearance of aggregates and improved synaptic function and behaviour [26].

In vitro, the conversion of soluble α-synuclein into amyloid fibrils typically occurs after a lag phase that is followed by a rapid increase in fibril elongation and is concentration-dependent [27]. This indicates that once a critical amount of α-synuclein amyloid precursors form stochastically, they act as “seeds” promoting the recruitment of α-synuclein monomer to the ends of these initial amyloidogenic units. The assembly of ɑ-synuclein into high molecular weight complexes is mediated by its central, hydrophobic sequence that is prone to aggregation. This sequence, is also termed the nonamyloid component of Aβ or NAC domain [28]. Therefore, at least in vitro*,* α-synuclein possesses properties that could explain its pathogenicity by a conformational templating mechanism. Accordingly, the A53T, H50Q and E46K α-synuclein mutations have been shown consistently to increase the rate of self-aggregation [27–31]. Duplication and triplication of the *SNCA* gene may also favour aggregation by increasing the concentration of assembly-competent conformers. On the other hand, not all mutations share this property; the A30P and G51D mutations impair the binding of α-synuclein to lipids or brain vesicles and decrease α-helical folding of its N-terminus [32, 33]. Therefore, in the crowded environment of the cell, assembly depends on additional factors such as altered interactions with membrane lipids.

**Fig. 1:**
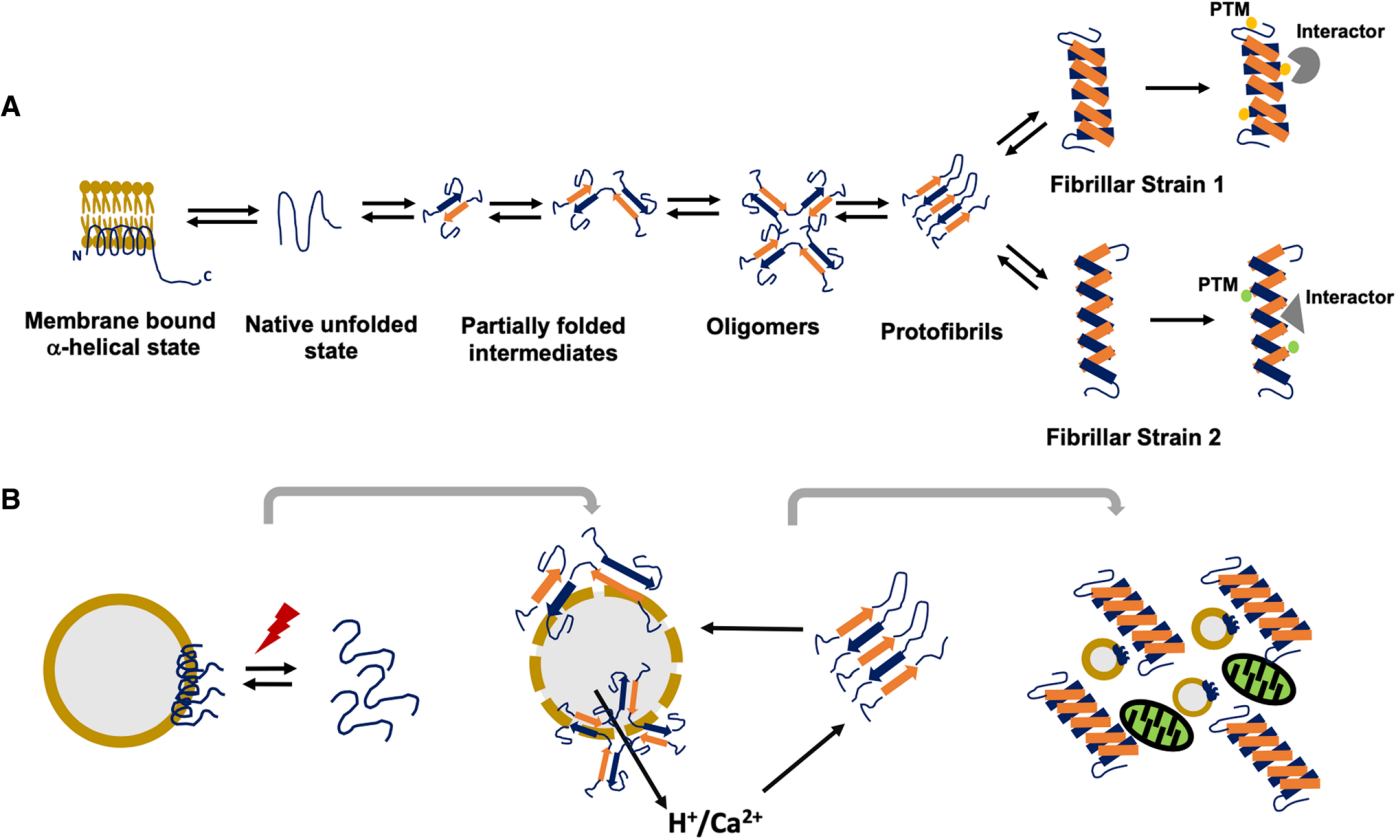
Bi-directional equilibrium between α-synuclein conformational states. **A** α-Synuclein acquires a partially folded α-helical structure when bound to lipid membranes but is natively unfolded in solution. Under favourable conditions, unfolding of the N-terminus and exposure of the NAC domain triggers oligomerisation via partially folded intermediates. Oligomers convert into β-sheet containing protofibrils and highly ordered cross-β-sheet fibrillar polymorphs (strains). Distinct amino acid side chains exposed on the surface of each strain may lead to differential post-translation modifications (PTM) or protein interactions. By differentially evading protective factors or disrupting functional protein complexes such interactions may explain variance in strain toxicity, cellular vulnerability and potentially disease severity. **B** Focal accumulation of α-synuclein on or around membranes due to impaired turnover, mutations, post-translational modifications or changes in lipid composition could initiate misfolding and assembly of toxic oligomers. Amyloidogenic oligomers disrupt membrane integrity, causing local changes in pH or Ca^2+^ levels that promote fibril formation and disruption of organelle function

Natively unfolded α-synuclein in solution, adopts an α-helical conformation in its N-terminal domain in the presence of membranes with acidic phospholipid headgroups and/or high curvature [34–36]. This interaction of α-synuclein with membranes may normally reduce misfolding into a β-sheet assembly [37] and/or promote physiological multimers [38, 39] that mediate its function in SNARE complex assembly and synaptic vesicle recycling [40]. On the other hand, the presence of lipids and detergents was also shown to increase the rate of α-synuclein fibril formation [41, 42]. A critical factor appears to be the lipid composition [43, 44] as well as the ratio of α-synuclein to lipid or detergent with lower concentrations of lipid driving and higher concentrations of lipid preventing aggregation [45]. This observation suggests that one mechanism by which lipids or lipid-like molecules may facilitate α-synuclein aggregation is by confining the protein to a small surface thereby increasing the local effective concentration to a critical threshold. This may also trigger the formation of helical intermediates of α-synuclein as suggested by NMR studies [46]. Additional factors are likely to promote aggregation-prone conformations of α-synuclein around membranous compartments such as focal changes in pH or Ca^2+^ concentration (Fig. 1). For example, Ca^2+^ binding to the C-terminus of α-synuclein may induce N-terminal unfolding and aggregation-prone conformations [47]. Low pH minimises the large net negative charge especially at the C-terminus, thereby decreasing charge-charge intramolecular repulsion, permitting hydrophobic interaction-driven collapse to a partially folded intermediate [48]. In this context, C-terminal truncation, e.g. by stress-induced activation of proteases, was also shown to accelerate aggregation [21].

Interestingly, α-synuclein aggregation on membranes has been shown to disrupt their integrity by different mechanisms [49, 50], which could in turn cause pH changes around endosomes, lysosomes or synaptic vesicles from leaked H^+^, triggering further aggregation. Once aggregated, α-synuclein interacts with transmembrane proteins on organelles such as SERCA on ER [51], TOM20 on mitochondria [52] and LAMP2A on lysosomes [53] impairing their function, thus setting up a pathogenic vicious circle that eventually disrupts synapses and neuronal function (Fig. 1B). The concept of α-synuclein aggregate initiation on or around membranes and its impact on organellar function is supported by the ultrastructure of Lewy bodies. Early electron microscopy showed that Lewy bodies have a dense osmiophilic core composed of neuromelanin, lipofuscin, mitochondria, dense core vesicles and endosomes surrounded by radially or haphazardly oriented fibrils [54]. This was confirmed more recently using correlative light and electron microscopy and tomography showing a crowded environment of membranes in Lewy bodies, including vesicular structures and dysmorphic organelles interspersed with fibrils [55]. A similar sequestration and disruption of organelles has been reproduced in a neuronal model of α-synuclein aggregation [56].

## α-Synuclein pathology progression involves cell non-autonomous mechanisms

The finding of neuronal α-synuclein inclusions in embryonic neural grafts 11–16 years after transplantation [57, 58] in patients, raised the possibility that aggregated α-synuclein from the host PD brain may have passed into the grafted cells, and templated the conversion of soluble α-synuclein similar to infectious cases of human prion diseases. These studies reinvigorated an earlier suggestion by Braak and colleagues, whose post-mortem neuropathological studies indicated that in the majority of cases, Lewy pathology evolves along interconnected brain networks, which begin in the dorsal motor nucleus of the glossopharyngeal and vagal nerves or the olfactory bulb and the anterior olfactory nucleus spreading rostrally [59]. In transgenic mice, human α-synuclein can transit to nerve cells grafted into the striatum [60]. Injection of recombinant or PD brain-extracted α-synuclein assemblies into the striatum or olfactory bulb of wild-type mice gave rise to α-synuclein inclusions and progressive neuronal loss [61–63]. These observations in mice have been replicated with injection of α-synuclein fibrils or Lewy bodies extracts into the brain of monkeys [64, 65]. Several mechanisms have been implicated in the internalisation of α-synuclein fibrils, principally via endocytosis [66], non-conventional pathways such as tunnelling nanotubes [67] or heparan sulfate proteoglycan mediated micropinocytosis [68]. It is likely that some mechanisms are cell-type specific or peculiar to a subcellular domain [68, 69] but the existence of a “receptor” for α-synuclein fibrils is controversial [70]. The strength of connections has emerged as a primary determinant of the spread of fibrillar forms of α-synuclein pathology when injected in different brain regions [71–73]. However, injections of fibrils in the pedunculopontine nucleus showed that the pattern of pathology was not a simple function of connectivity or synaptic coupling [74]. Therefore, although synaptic connectivity constrains the spread of α-synuclein pathology in the brain, other factors play a critical role in determining its pattern, severity or temporal evolution. In this respect, a key determinant is the cell type-specific expression level of endogenous α-synuclein [71, 73, 75]. Other factors include the axonal arborisation and metabolic stress, which is similarly extensive for dopaminergic and cholinergic neurons that are especially vulnerable [76] as well as the activation state of surrounding microglia and potentially other glial cells [77, 78].

Lewy bodies in PD patients are also found in the enteric nervous system raising the possibility that the spread of α-synuclein aggregates also occurs between the gastrointestinal system and the brain. In support of a gut-to-brain spreading, vagotomy has been associated with a reduced risk of PD [79]. In animals, all α-synuclein species (monomers, oligomers and fibrils) when injected in the intestine were actively transported to the dorsal motor nucleus of the vagus by both slow and fast axonal transport [80]. Fibril injection in the duodenal muscularis layer triggered the spread of pathogenic α-synuclein, as assessed by its phosphorylation at serine 129, first in the dorsal motor nucleus and subsequently more rostrally, and was prevented by truncal vagotomy or injection in α-synuclein knockout mice [81]. On the other hand, injection of Lewy body extract in baboon monkeys showed that the progression of α-synuclein pathology was either caudo-rostral or rostro-caudal but not found in the vagal nerve, supporting a systemic route for long-distance bidirectional spread between the enteric and the central nervous systems [82]. One potential mechanism is the release of α-synuclein in neuronally derived extracellular vesicles [83] which is increased in PD patients even in the prodromal phase [84]. Interestingly, systemic injection of fibrils into the circulation also induces brain pathology in rodents [85] and α-synuclein aggregates have been detected in the serum of PD patients [86] but whether the latter are seeding-competent remains to be seen.

## The precise structure and size of the toxic species in PD remains unresolved

It is currently unknown which α-synuclein assemblies are the most relevant to the human condition. It is well established that a β-sheet conformation is an essential element of seeding and toxicity. For example, single molecule techniques have revealed that different types of oligomers may assemble during the formation of α-synuclein fibrils, but only those that are proteinase K resistant, i.e. containing β-sheet conformation, are damaging to cells [87, 88]. Kinetically trapped β-sheet oligomers were shown to contain approximately 30 monomers [89]. The minimum number of α-synuclein molecules for stable fibril generation was recently estimated to be approximately 70 monomers, giving a size of approximately 40 nm [90]. A bidirectional equilibrium exists between oligomers and higher molecular weight fibrillar assemblies [87, 91] raising the possibility that cellular toxicity and seeding competency are mediated by conformers or assemblies of different size along this continuum. Studies in vitro showed that the fibrillation kinetics vary between different α-synuclein mutants, whereas the steady-state population of oligomeric intermediates is a shared property, suggesting that this may be the toxic species [91]. Experiments in animals using a lentiviral expression of α-synuclein mutants that promote either oligomer or fibril formation showed that the most severe dopaminergic loss in the substantia nigra was observed with those α-synuclein variants that form oligomers [92]. On the other hand, when injected into animals, fibrils but not oligomers were able to seed aggregation and cause toxicity [85]. Although vis-à-vis comparison between these two different models is problematic, one potential unifying explanation of these disparate findings is that oligomers per se do not propagate, but are generated during the assembly or disassembly of seeding-competent fibrils inside neurons, as has been shown in vitro [87], and constitute the primary drivers of neuronal damage. Whether such oligomers are generated or stabilised intraneuronally, e.g. by post-translational modifications or incomplete disaggregation/degradation remains to be seen. For example, the vulnerability of dopaminergic neurons has been partly ascribed to oxidation of dopamine in the cytosol, forming reactive quinones and partially reduced oxygen species that stabilise potentially toxic oligomers of α-synuclein [93, 94]. Phosphorylation at Serine 129 is present in > 90% of α-synuclein isolated from the human brain aggregates [95] but the effect of this abundant modification on assembly is controversial [95–97] and may also occur after aggregation.

Another important consideration is that α-synuclein assembles into structurally distinct fibrillar polymorphs or strains. This has been demonstrated by generating strains de novo using different experimental conditions [98, 99] and by the cryo-electron microscopic characterisation of either sarkosyl-insoluble brain-extracted fibrils or fibrils amplified in the presence of brain homogenate or CSF from different α-synucleinopathies [100–102]. It remains to be determined which structure most accurately represents the seeding-competent assemblies that form inside intact neurons in the human condition and what is the influence of detergents or the extraction/amplification process on their structure. Nevertheless, the generation and characterisation of these fibrillar α-synuclein strains led to the hypothesis that they are in part responsible for the heterogeneous clinical manifestation of α-synucleinopathies. This is supported by findings in human iPSC-derived dopaminergic neurons [75] and animal models [103] showing that strains amplified in a pure form from MSA brain homogenate are more toxic than strains amplified from Lewy body disease brain homogenate. Similarly, intracerebral injection of MSA brain extract into transgenic mouse brains led to abundant α-synuclein inclusions and neurodegeneration that was not observed to the same extent with PD brain extract [104, 105]. These findings in experimental models reflect the more aggressive nature of MSA in patients which unlike PD causes death within 7–10 years from onset.

Why these strains exhibit different toxicities is not fully understood. Although a critical level of aggregation is necessary for neuronal loss, which in turn depends on the levels of monomeric α-synuclein [75], additional mechanisms are at play. One explanation is the differential interactions of α-synuclein strains with the cellular proteome due to different amino acid stretches, in particular their side chains, that are exposed on the surface of each conformer (Fig. 1A). In this way, strains could differentially disrupt critical protein functions or evade homeostatic defences and/or be subjected to different post-translational modifications. We recently used proximity-dependent biotin identification to label interacting proteins within ~ 10 nm radius of α-synuclein aggregation in cells seeded with de novo-generated or brain-amplified fibrils. We found that α-synuclein interacts with approx. 1000 cellular proteins during assembly but only 56 proteins were differentially interacting between strains including the PD-associated protein DJ-1 [75]. Loss-of-function mutations in DJ-1 cause autosomal recessive PD with Lewy bodies [106] and CRISPR/Cas9 knockout of DJ-1 in our human iPSC-derived model increased seeded aggregation and aggregate-induced neuronal death [75] potentially via loss of its deglycase activity [107]. Glycation may block the ubiquitination of exposed lysine residues on misfolded α-synuclein such as the N-terminal lysine 12 [75, 108–110], in this way preventing its efficient degradation. Fibrils of α-synuclein are also subjected to disaggregation by the chaperone HSC70 and members of the HSP90 family, mediated by the recognition of a canonical motif in the N-terminus of α-synuclein [111], which is enriched in lysine residues. It is, therefore, possible that in the human brain, the fold of certain α-synuclein strains confers selective advantage by evading protective responses to become dominant and pathogenic whereas interactions with protective factors, such as DJ-1, mitigate their toxicity in the most resilient cells. Accordingly, in many brain regions of experimental animals α-synuclein pathology induced by fibrils was transient, demonstrating that certain cell types are more efficient in eliminating α-synuclein aggregates [73, 74]. Strains may also “evolve” differently depending on cell-type-specific proteostatic pressures: for example, oligodendrocytes but not neurons were shown to transform misfolded α-synuclein into an MSA-like strain [105]. Differential interactions are expected not only to imbalance proteostasis but also to sequester other proteins into non-functional states. Distinct fibrillar α-synuclein polymorphs bind to and cluster differentially at the plasma membrane in both primary neuronal cultures and organotypic hippocampal slice cultures from wild-type mice causing differential synaptic redistribution of α3-Na^+^/K^+^-ATPase and certain synaptic receptors [112].

## Translation of α-synuclein aggregation studies into clinical outcomes

The fundamental insights into the mode of assembly and trans-neuronal spread of α-synuclein provided a rationale for novel therapeutic approaches. Both active and passive immunotherapies have been extensively studied and shown to reduce α-synuclein pathology in animal models [113]. More recently, small molecule inhibitors of aggregation showed good efficacy in pre-clinical models [114, 115]. Once further delineated, generic mechanisms of uptake or clearance of pathogenic assemblies may also be suitable for therapeutic targeting. Phase II clinical trials in humans so far have been performed with two anti-α-synuclein antibodies: Prasinezumab (PRX002), a humanised version of the mouse monoclonal antibody 9E4 [116] that binds to the C-terminus, and Cinpanemab (BIIB054), a human derived antibody that binds to the N-terminus of α-synuclein [117]. Both antibodies bind to aggregated α-synuclein but it is unclear whether either binds to disease-relevant species (e.g. seeding-competent assemblies) that are thought to be responsible for the progression of the pathology. Although treatment with both antibodies missed the primary efficacy outcomes, prasinezumab significantly slowed the decline on the motor examination (UPDRS Part III) and the digital motor score by 25–30%, and participants with more severe and faster-progressing symptoms benefitted the most from treatment. Based on these encouraging findings, a Phase IIb study (PADOVA) in PD patients with more advanced symptoms was started and will run through to 2023.

A major limitation in the development of these therapies is the lack of biomarkers that measure target engagement in the CSF or brain. Currently there is no PET ligand available for α-synuclein aggregates but the resolution of their structure by cryo-electron microscopy may facilitate this urgently needed biomarker. The concept of self-templating assembly of α-synuclein has led to the adaptation of assays previously used for prions (RT-QuIC or PMCA) to measure α-synuclein seeding when incubated with CSF [118, 119] and more recently peripheral tissue homogenates [120]. α-Synuclein seeding assays in the CSF showed sensitivities and specificities for PD diagnosis at 80–90% with a high degree of overlap between the two assay types [121]. Interestingly, RT-QuIC also identified individuals at risk of developing PD and dementia with Lewy bodies, indicating that seeding assays may be useful in detecting prodromal α-synucleinopathies [122]. Based on the kinetics of aggregation in the PMCA assay, it was shown that PD could also be distinguished from MSA with an overall accuracy of 95% [100]. Therefore, once further validated and standardised, RT-QuIC or PMCA could be used to stratify patients that are most likely to benefit from specific immunotherapies e.g. based on the binding affinity of antibodies to patient-specific seeds, assuming that CSF seeds resemble those in brain, or to monitor treatment response. For this purpose, further development is required to achieve a quantitative readout, ideally in an easily accessible peripheral source of tissue or biofluid.

## Conclusions

Since the original discovery of α-synuclein as the main component of Lewy bodies in sporadic PD and the identification of *SNCA* mutations in rare familial forms, significant progress has been made in understanding its role in human disease. Early studies in transgenic or viral overexpression animal models demonstrated that pathogenicity arises from a toxic-gain-of function of misfolded α-synuclein that is most likely initiated at the synapse. Multiple lines of evidence indicate that toxicity is mediated by aggregate-induced interference with synaptic vesicle recycling and organelle function. Modelling of aggregation in vitro revealed a self-templating mode of assembly, suggesting that a similar mechanism may account for the spread of the pathology and the progressive march of clinical symptoms in patients. This has been demonstrated in a number of animal models following a single injection of synthetic or brain-derived fibrils. At least in animals, the pattern of spread is determined by neuronal connectivity and levels of expression of endogenous α-synuclein. Additional cellular factors, such as the interaction of α-synuclein with protein partners or membrane lipids may influence the emergence of distinct conformers or strains, which could at least partly explain the clinical heterogeneity of α-synucleinopathies. Although it is widely accepted that a β-sheet conformation is an essential component of pathogenic α-synuclein assemblies, the remarkable conformational plasticity of α-synuclein and resulting conformation ensembles has made it difficult to precisely define the structure that is most relevant to the human condition. Extensive neuropathological observations in post-mortem PD brains have lend credence to the concept of propagation of such assemblies along connected networks but it is unclear whether and to what extent this occurs in living patients. It is conceivable that PD is the result of multiple stochastic aggregation events due to impaired protein or lipid homeostasis along interconnected neuronal networks or their supporting glial cells with limited capacity to spread beyond a single synapse. It is also possible that in the human brain there are variants of the dominant strain across brain regions and/or patients. If this is true, then a single immunotherapy or anti-aggregation therapy will never be efficacious across the whole disease spectrum and a molecular approach to patient classification will be required to identify those most likely to benefit, as is currently done with targeted therapies in cancer. In this respect, the application of cryo-electron microscopy and modelling in human neurons and animals have the potential to introduce a step-change in the way we stratify and eventually treat α-synucleinopathies with precision diagnostics and therapeutics, ideally introduced in the prodromal phase of carefully selected at-risk individuals.

## Data Availability

Not applicable.
